# Carbon Fibers Encapsulated with Nano-Copper: A Core–Shell Structured Composite for Antibacterial and Electromagnetic Interference Shielding Applications

**DOI:** 10.3390/nano9030460

**Published:** 2019-03-19

**Authors:** Yue Jiao, Caichao Wan, Wenbo Zhang, Wenhui Bao, Jian Li

**Affiliations:** 1 Material Science and Engineering College, Northeast Forestry University, Harbin 150040, China; yjiao123@126.com (Y.J.); 474007776@163.com (W.Z.); baowenhuinefu@163.com (W.B.); 2 College of Materials Science and Engineering, Central South University of Forestry and Technology, Changsha 410004, China

**Keywords:** carbon fibers, core–shell structure, magnetron sputtering, antibacterial materials, electromagnetic interference shielding

## Abstract

A facile and scalable two-step method (including pyrolysis and magnetron sputtering) is created to prepare a core–shell structured composite consisting of cotton-derived carbon fibers (CDCFs) and nano-copper. Excellent hydrophobicity (water contact angle = 144°) and outstanding antibacterial activity against *Escherichia coli* and *Staphylococcus aureus* (antibacterial ratios of >92%) are achieved for the composite owing to the composition transformation from cellulose to carbon and nano-size effects as well as strong oxidizing ability of oxygen reactive radicals from interactions of nano-Cu with sulfhydryl groups of enzymes. Moreover, the core–shell material with high electrical conductivity induces the interfacial polarization loss and conduction loss, contributing to a high electromagnetic interference (EMI) shielding effectiveness of 29.3 dB. Consequently, this flexible and multi-purpose hybrid of nano-copper/CDCFs may be useful for numerous applications like self-cleaning wall cladding, EMI shielding layer and antibacterial products.

## 1. Introduction

Recently, rapid consumption of non-renewable resources (like petrochemical resources) and increasing seriousness of environmental pollutions have prompted researchers to pay more attention to the utilization of green and renewable biomass resources. Thus, biomass-based functional materials are attracting increasing interest from research and industrial circles. For instance, some biomass materials (such as wheat straw, wood and bamboo fibers) are directly combined with nanomaterials for specific applications like water purification and energy storage [1,2,3,4,5]. Biomass materials can also be transformed into their corresponding carbon counterparts for the development of various electroresponse products [6,7]. Moreover, some nano-components or novel reconstituted materials from biomass resource (like 1D cellulose nanofibrils [8], 2D cellulose films [9,10] and 3D cellulose hydrogel or aerogels [11,12,13,14]) are used as templates to support multifarious guest substances for the creation of novel and eco-friendly functional composites [15]. In the process of preparation, numerous physicochemical methods (e.g., hydrothermal method [16], vapor phase polymerization [17], electrodeposition [18,19] and atomic layer deposition [20,21]) are involved. However, some studies generally involve high consumption of energy and chemicals during the separation/disassembly of raw materials and complicated or low-precision synthetic methods, which seriously restrict the practical applications and bulk production of these biomass-based functional materials. Therefore, it is of significance to screen easily available and cheap raw materials as well as mild and scalable synthetic methods.

Amongst a variety of physicochemical techniques, magnetron sputtering is a powerful sputtering-based ionized physical vapor deposition technique and is already making its way to industrial applications [22]. Its deposition is achieved by rapidly colliding ionized inert gas atoms (commonly Ar) with the surface of negatively biased target under high electric field and thus inducing the ejection (sputtering) of atoms which condenses on a substrate and eventually generates a membrane [23]. Magnetron sputtering has numerous advantages, like high purity, high adhesion, high deposition rate, excellent uniformity on large-area substrates, ease of automation, ease of sputtering any metals, alloys or compounds and extensive applicability (like heat-sensitive substrates) [24]. The deposition of metallic materials by magnetron sputtering may improve the electrical conductivity, hydrophobicity and electroresponse of biomass materials, thus expanding their potential application areas [25,26]. Besides, compared to other methods that deposit metals onto biomass materials (like electrodeposition, electroless plating and chemical reduction method), the magnetron sputtering method has a stronger capability to accurately control the thickness, homogeneousness and purity of deposited layer [27]. Therefore, magnetron sputtering is an ideal technique for the surface modification of natural biomass.

In this work, we adopted a low-cost and widely available biomass material (i.e., cotton cloth) as raw materials. For the purpose of extending the application scopes, the cotton fibers were firstly pyrolyzed into a conductive and hydrophobic cotton-derived carbon fibers (coded as CDCFs). A thin layer of nano-copper was then deposited on the surface of CDCFs via the facile magnetron sputtering, resulting in the generation of core–shell structured composite. As a typical application example, the nano-Cu/CDCFs composite serves as a multi-purpose electromagnetic interference (EMI) shielding material with favorable flexibility, antibacterial activity and hydrophobic nature. In addition, the synergistic effects of nano-Cu and CDCFs compositions on the above properties of the composite were analyzed.

## 2. Experimental Section

### 2.1. Materials

Common old jean (100% cotton) was employed as the feedstock of CDCFs after being washed repeatedly with distilled water and ethyl alcohol and then dried at room temperature. A Cu target (purity: 99.99%) with a diameter of 50 mm was purchased from Shenyang Kejing Auto-instrument Co., Ltd., Shenyang, China. *Escherichia coli* (*E*. *coil*, ATCC 25922) and *Staphylococcus aureus* (*S. aureus*, ATCC 6538) were supplied by Guangdong Detection Center of Microbiology, Guangzhou, China. Other chemicals were provided by Kemiou Chemical Reagent Co. Ltd. (Tianjin, China) and used as received.

### 2.2. Preparation of Nano-Cu/CDCFs Composite

The preparation of nano-Cu/CDCFs composite is primarily based on two processes, i.e., pyrolysis and magnetron sputtering. First, the clean and dried jeans were transferred into a tubular furnace for pyrolysis under the protection of nitrogen. The sample was heated to 1000 °C at a heating rate of 5 °C min^–1^, and this temperature was maintained for 1 h to allow for complete pyrolysis; subsequently, the furnace decreased naturally to the room temperature and the following CDCFs were obtained. Second, the nano-Cu shell was deposited on the surface of CDCFs using magnetron sputter deposition technology with a DC sputter source (VTC-600-2HD, Shenyang Kejing Auto-instrument Co., Ltd., Shenyang, China), where the CDCFs and Cu target were placed on the anode and cathode with a distance of 60 mm between them, respectively. Regarding the sputtering process, we firstly pumped the chamber to a pressure of 3 × 10^–3^ Pa and then Argon was used as a sputtering gas and slowly introduced to the chamber with a flow rate of 11 sccm. With a target power of 100 W and rotating speed of 20 rpm, a homogeneous sputtering of Cu was achieved on the surface of CDCFs. With 40 °C maximum due to the water-cooling, the thermal stress of the substrate is on a very low level contributing to preventing the deformation and diffusion movement of the deposited Cu. The deposition thickness of Cu was set as 50 nm and the deposition was performed twice on the two sides of carbon cloth.

### 2.3. Characterizations

Morphology observations were performed on a scanning electron microscope (SEM, Hitachi S4800, Tokyo, Japan) equipped with an energy dispersive X-ray (EDX) detector. Crystal structure was analyzed by X-ray diffraction (XRD, Bruker D8 Advance TXS, Karlsruhe, Germany) with Cu Kα (target) radiation (*λ* = 1.5418 Å). The scan rate and scan range were set as 4° min^–1^ and 10–80°, respectively. Water contact angle (WCA) tests were performed on a contact angle analyzer (JC2000C, Zhongchen Digital Technic Apparatus Co., Ltd., Shanghai, China). Electrical conductivity was tested using a four-point probe resistivity/square resistance tester (KDB-1, Kunde Technology Company Ltd., Guangzhou, China).

### 2.4. Antibacterial Activity Studies

Antibacterial activity studies were conducted using a shake flask method [28]. *E. coli* and *S. aureus* were used as the models of Gram-negative and Gram-positive bacteria for the tests. For preparing bacteria suspensions, the bacteria were grown in Luria Broth (LB) growth solutions for 18 h at 37 °C. A colony was lifted off with a platinum loop, placed in 30 mL of nutrient broth, and incubated with shaking for 18 h at 37 °C. After washed twice with phosphate buffer saline (PBS, pH = 7.4), they were resuspended in PBS to yield 1.0–1.5 × 10^5^ colony forming unit (CFU) mL^−1^. By measuring the absorbance of cell suspension, the bacterial cell concentration can be estimated [29]. To evaluate the antimicrobial properties of the cotton fibers, CDCFs and nano-Cu/CDCFs, 1 × 1 cm^2^ of the sample was immersed into a falcon tube containing 5.0 mL of 1.0 × 10^−3^ M PBS culture solution with a cell concentration of 1.0–1.5 × 10^5^ CFU mL^−1^. The falcon tube was then shaken at 200 rpm on a shaking incubator at 25 °C for 24 h. After shaking vigorously to detach adhered cells from the sample surfaces, the solution was serially diluted, and then 0.1 mL of each diluent was spread onto the agar plates. Viable microbial colonies were counted after incubating the plates for 18 h at 37 °C. In addition, a blank control experiment was also conducted following the same method while any tested materials were not added into the falcon tube containing PBS culture solution and bacterial cell.

### 2.5. EMI Shielding Effectiveness Studies

EMI shielding effectiveness was measured with the samples of dimension of 22.9 mm × 10.2 mm × 2 mm to fit waveguide sample holder using a PNA-X network analyzer (N5244a, Agilent Technologies, Palo Alto, State of California, USA) at the frequency range of 8.2–12.4 GHz (X-band). *S*-parameters connect the input and output circuit quantities using the reflection and transmission parameters normally adopted in microwave analysis. By means of such parameters, it is possible to determine the EMI shielding effectiveness due to reflection or absorption.

## 3. Results and Discussion

### 3.1. Schematic Diagram for Preparation of Nano-Cu/CDCFs Composite

**Figure 1:**
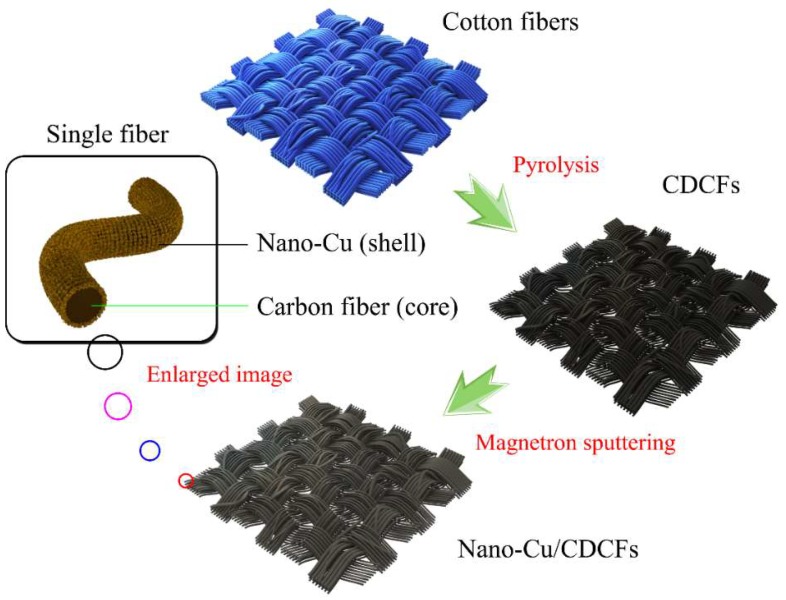
Graphical illustration of the design concept of the core–shell structured nano-Cu/CDCFs composite.

For the sake of seeking easily available and cheap biomass feedstock and developing mild and scalable methods for the synthesis of novel and high-performance functional products, as illustrated in Figure 1, we chose disused jean cloth (100% cotton) as raw material and its main composition (namely cellulose) is easily transformed into the corresponding carbon material possessing new functions (like electrical conduction and hydrophobicity). The following magnetron sputtering process was conducted through the atom ejection of target materials due to the collision of high-speed Ar^+^, resulting in the deposition of Cu on the surface of fibers. Therefore, this simple and easily scalable two-step method could generate the core–shell structured nano-Cu/CDCFs composite.

### 3.2. Morphology Observations, Elemental Analysis and Crystal Structure

**Figure 2:**
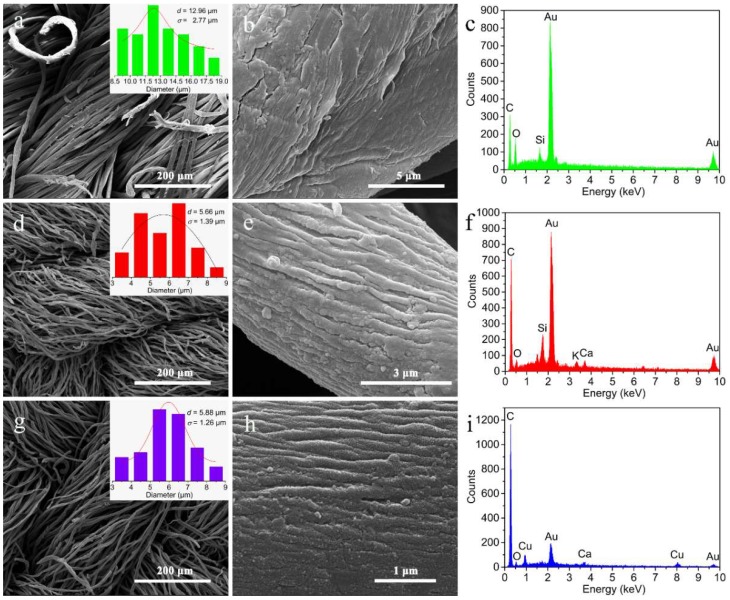
SEM images and EDX patterns of the cotton fibers (**a**–**c**), CDCFs (**d**–**f**) and nano-Cu/CDCFs (**g**–**i**): (**a**,**d**,**g**) distribution state of fibers and insets show the corresponding diameter distribution of fibers; (**b**,**e**,**g**) surface observation of single fiber; (**c**,**f**,**i**) EDX patterns.

The changes of morphology and elemental compositions derived from the treatments of pyrolysis and magnetron sputtering were analyzed by SEM and EDX. By comparing Figure 2a,d, we can find that the pyrolysis causes a reduction in the average size of fibers from 12.96 μm (cotton fibers) to 5.66 μm (CDCFs), while the fibers still tightly intertwine with each other after the pyrolysis ensuring the structural integrity of carbon cloth. Moreover, the fiber surface becomes rougher (Figure 2b,e) after the pyrolysis owing to the thermal decomposition of most oxygen-containing compositions. Also, the significantly increased C/O ratio from 1.3 to 8.1 based on the EDX patterns (Figure 2b,e) indicates the transformation from cellulose to carbon. Through the subsequent magnetron sputtering, the diameters and entangled state of fibers are almost unchanged (Figure 2g), while the surface of CDCFs was coated with many particles with a size of dozens of nanometers (Figure 2h), resulting in the formation of a core–shell structured composite, i.e., the CDCF serves as the core part and the superficial nano-layer acts as the sheath part. The thickness of nano-Cu/CDCFs is about 0.2 mm and its density is around 0.317 g cm^−3^, similar to that of pure CDCFs (ca. 0.315 g cm^−3^). Moreover, Cu signals were detected in the EDX pattern of the composite (Figure 2i), revealing that Cu element is one of the main elemental compositions of nano-layer. These results demonstrate that the two-step method successfully generated the core–shell structured composite. In addition, Au peaks in these EDX patterns were originated from the coating layer used for electrical conduction during the SEM observation.

**Figure 3:**
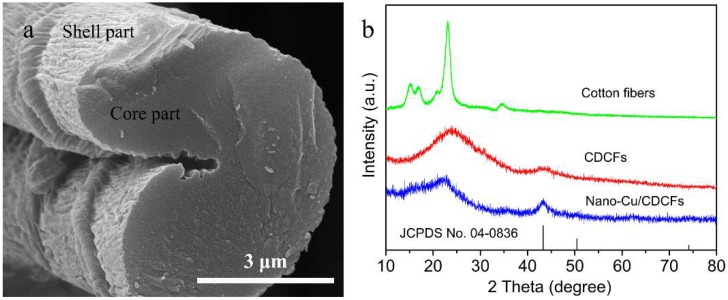
(**a**) cross-section SEM image of nano-Cu/CDCFs; (**b**) XRD patterns of the cotton fibers, CDCFs and nano-Cu/CDCFs, and the bottom line is the standard JCPDS card no. 04-0836 for Cu.

The cross-section SEM image of nano-Cu/CDCFs is exhibited in Figure 3a, where a core–shell structure can be clearly identified. XRD analysis was carried out to study the changes of crystal structure before and after the pyrolysis and magnetron sputtering. From Figure 3b, cotton fiber (mainly consisting of cellulose) displays a typical cellulose I crystal structure with peaks at 15.3°, 17.0°, 23.2° 20.9° and 34.6°, corresponding to the planes of (101), (10$\bar{1}$), (021), (002) and (040), respectively [30]. These characteristic peaks disappear after the pyrolysis while two broad peaks centered at around 24.1° and 43.3° appear in the XRD pattern of CDCFs, which are related to the (002) and (100) planes of graphite and suggest the generation of amorphous carbon [31]. The result agrees well with that of EDX analysis. After the deposition of Cu nanoparticles by magnetron sputtering, a new peak at 43.3° is detected and related to the (111) plane of Cu (JCPDS No. 04-0836). In addition, the (002) diffraction shifts towards a lower angle after the magnetron sputtering, indicative of a decrease in the order of crystallinity in carbon materials [32].

### 3.3. Hydrophobic Property and Antibacterial Activity

**Figure 4:**
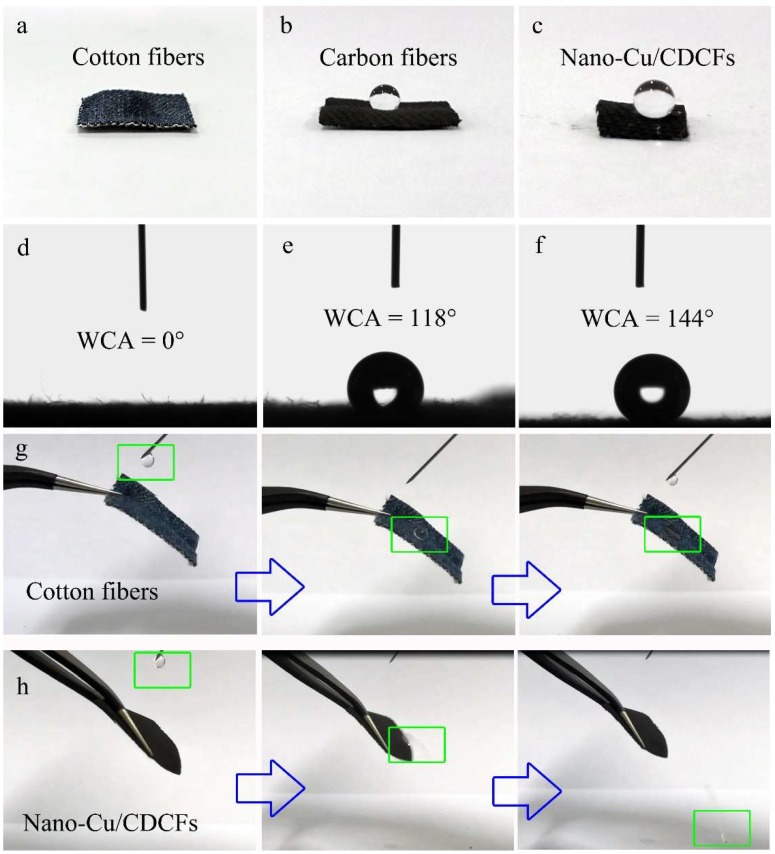
Hydrophobic property and WCA tests of the cotton fibers (**a**,**d**), CDCFs (**b**,**e**) and nano-Cu/CDCFs (**c**,**f**), respectively. Photographs of water drop contacting the surfaces of the cotton fibers (**g**) and nano-Cu/CDCFs (**h**).

Hydrophobic property plays a crucial role in the self-cleaning ability of materials [33]. The influences of the treatments of pyrolysis and magnetron sputtering on the hydrophobic property were studied by WCA tests. As shown in Figure 4a and d, the cotton fibers can readily absorb water drop once the drop contacts its surface, indicative of favorable hydrophilicity of the cotton. By contrast, the pyrolyzed carbon fibers can stably support a water drop on its surface with a WCA value of 118° (Figure 4b,e), suggesting the formation of hydrophobicity owing to the pyrolysis. After the coating of nano-Cu, the core–shell material shows an improved hydrophobic property with a higher WCA value of 144° (Figure 4c,f). Furthermore, comparing Figure 4g,h, it is clear that the water drop quickly tumbles from the surface of nano-Cu/CDCFs as soon as the drop touches the surface, further demonstrating its excellent water repellency. This dropping process of water mixed with various smudges (like dust and mucus) rises an important self-cleaning function. In addition, we also calculated some characteristic parameters including the work of adhesion (*W*_a_), the coefficient of spreading (*S*) and the work of wetting (*W*_w_) via the equations, i.e., *W*_a_ = *γ_LG_*(1 + cos*θ*), *S* = *γ_LG_*(cos*θ* − 1) and *W*_w_ = *γ_LG_*cos*θ* (*γ_LG_* is the interfacial tension between liquid and gas, *θ* is the water contact angle) [34], respectively. In this paper, *θ* is 144° and *γ_LG_* is 72.75 mN m^–1^; thus, *W*_a_, *S* and *W*_w_ are calculated as 13.9, −131.6 and −58.9 mN m^–1^, respectively. The negative values of *S* and *W*_w_ reveal that the water cannot be spread on the surface of nano-Cu/CDCFs, i.e., excellent hydrophobicity.

**Figure 5:**
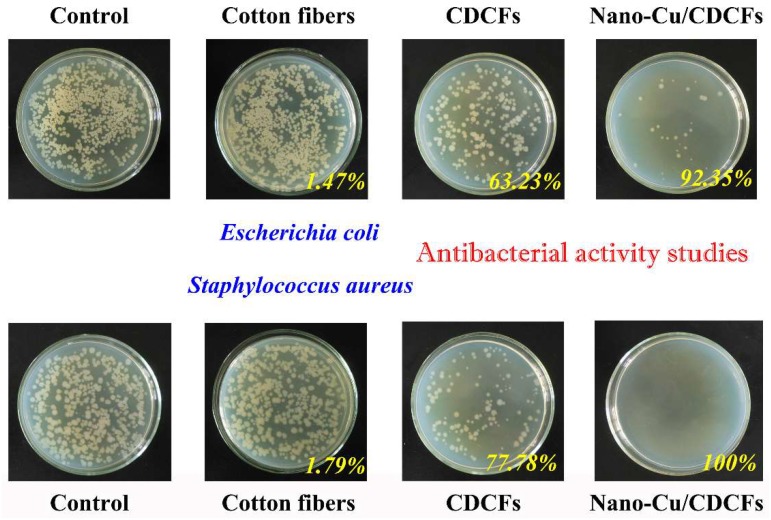
Antibacterial activity studies of the cotton fibers, CDCFs and nano-Cu/CDCFs using a shake flask method.

The discovery of antibacterial effects of Cu can be traced back to ancient civilizations that used to employ different forms of Cu compounds to treat several afflictions and to maintain hygiene. However, the reports on the antibacterial properties of Cu-containing nanomaterials are not abundant. Herein, the antibacterial activity of the cotton fibers, CDCFs and nano-Cu/CDCFs was tested via a shake flask method. *E. coli* and *S. aureus* were chosen as the models of Gram-negative and Gram-positive bacteria for the tests. As shown in Figure 5, the surface concentrations of live *E. coli and S. aureus* on the cotton fibers (ca. 6.7 × 10^10^ and 5.5 × 10^10^ CFU cm^−2^) are close to those of blank control (ca. 6.8× 10^10^ and 5.6× 10^10^ CFU cm^−2^), indicative of a negligible antibacterial activity of the cellulose material (i.e., cotton fibers). The phenomenon is consistent with that of our previous study [35]. Through introducing CDCFs, the surface concentrations of live *E. coli* and *S. aureus* decline to 2.5 × 10^10^ and 1.2 × 10^10^ CFU cm^−2^, respectively. The introduction of nano-Cu/CDCFs further reduces the concentrations to 5.2 × 10^9^ and 0 CFU cm^−2^, respectively, revealing an excellent antibacterial activity of nano-Cu/CDCFs with 92.35% and 100% of antibacterial ratios for *E. coli* and *S. aureus*. The origins of the antibacterial activity of nano-Cu/CDCFs may mainly be concluded to the two points: (1) the interaction of nano-Cu with sulfhydryl groups of enzymes is one of the possible protein oxidation routes resulting in the generation of oxygen reactive radicals that eventually causes irreparable damage like oxidation of proteins, cleavage of DNA and RNA molecules, and membrane damage due to lipid peroxidation [36,37]; (2) the direct cell contact with CDCFs may impact cellular membrane integrity, metabolic activity and morphology of bacteria [38].

### 3.4. EMI Shielding Properties

Nowadays, the widespread use of high-power machines (such as base station, radar and some household appliances like induction cooker) has caused serious electromagnetic pollution, which has a strong adverse impact on the human health and normal operation of equipment. Especially, electromagnetic pollution has been listed as the fifth major pollution sources on the earth, following atmospheric pollution, water pollution, solid waste pollution and noise pollution. Therefore, it is urgent to develop high-performance, cheap and easily produced EMI shielding products.

EMI shielding property is evaluated by shielding effectiveness expressed in decibels (dB) over the frequency range of 8.2–12.4 GHz (X-band). A higher decibel level reveals less energy transmitted through shielding materials. The total shielding effectiveness (*SE*_total_) can be expressed as [39]:

(1) $$SE_{\mathrm{total}}(dB)=10\log\frac{P_{i}}{P_{t}}=SE_{A}+SE_{R}+SE_{M},$$

where *P*_i_ and *P*_t_ are the incident and transmitted electromagnetic power, respectively. *SE*_R_ and *SE*_A_ are the shielding effectiveness from reflection and absorption, respectively. *SE*_M_ is multiple reflection effectiveness inside the material, which can be negligible when *SE*_total_ >10 dB. In addition, *SE*_R_ and *SE*_A_ can be described as [40]:

(2) $$SE_{R}=-10\log(1-R),$$

(3) $$SE_{A}=-10\log\left[T/(1-R)\right],$$

where *R* and *T* are reflected power and transmitted power, respectively.

**Figure 6:**
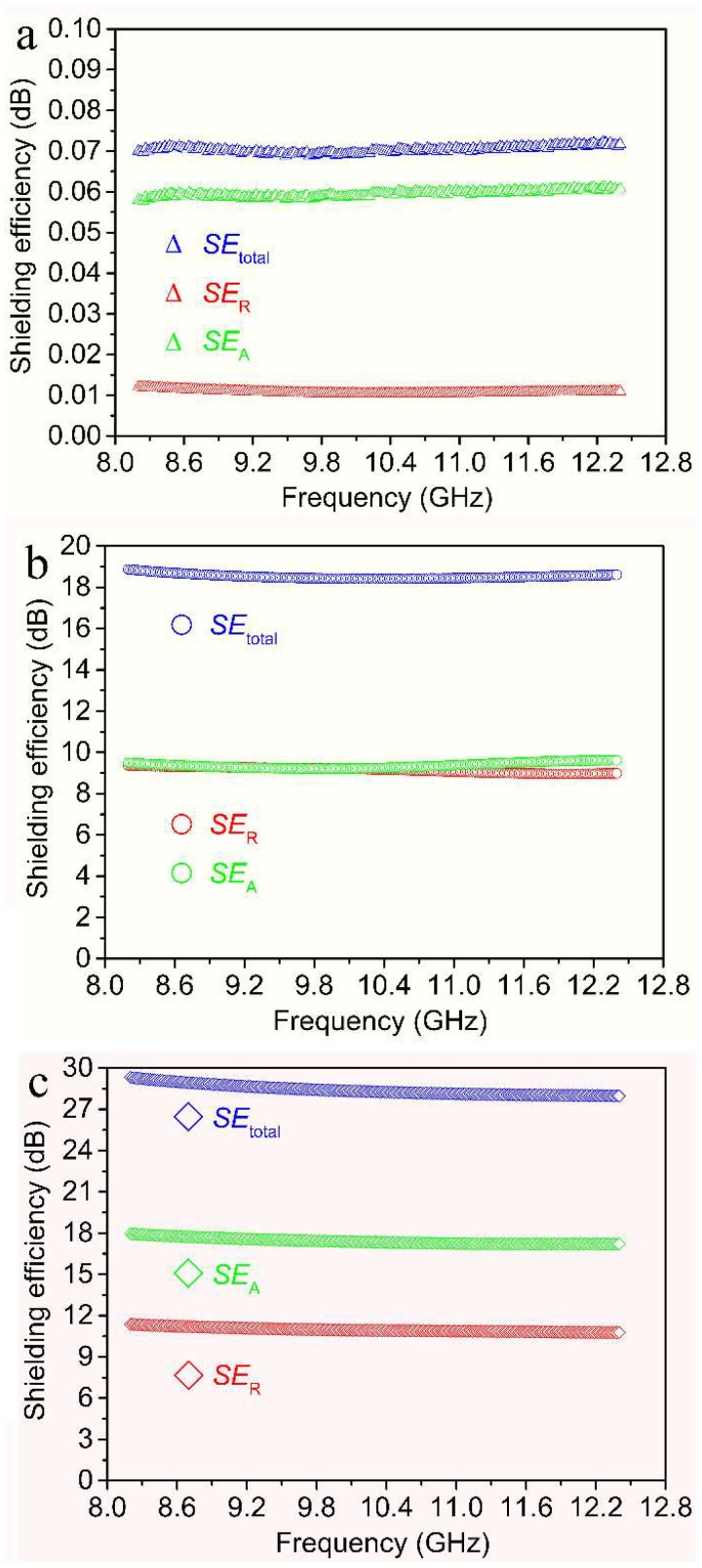
EMI shielding effectiveness (including *SE*_total_, *SE*_R_ and *SE*_A_) of (**a**) the cotton fibers, (**b**) CDCFs and (**c**) nano-Cu/CDCFs, respectively.

The shielding effectiveness of the cotton fibers, CDCFs and nano-Cu/CDCFs within the frequency scope of 8.2–12.4 GHz is presented in Figure 6. As seen in Figure 6a, the shielding effectiveness of the cotton fibers is negligible. The low *SE*_total_ value of 0.7 dB is ascribed to its ignorable magnetic permeability and electrical conductivity which are both decisive for EMI shielding effectiveness. In addition, CDCFs exhibit an obviously higher electrical conductivity of 4.57 S cm^–1^ than that of the cotton fibers (<5 × 10^–6^ S cm^–1^). As a result, the maximum *SE*_total_ value of CDCFs can reach 18.9 dB (Figure 6b), close to the requirement for the commercial EMI shielding applications (>20 dB). Moreover, the contributions from *SE*_R_ and *SE*_A_ account for 50.3 and 49.7% at 8.2 GHz, respectively, indicating that the EMI shielding action due to absorption is close to that due to reflection. The introduction of nano-Cu leads to the increase of electrical conductivity of nano-Cu/CDCFs to 20.3 S cm^–1^. Moreover, the surface resistivity of CDCFs and nano-Cu/CDCFs is calculated as ~10.9 Ω sq^–1^ and ~2.5 Ω sq^–1^, respectively, according to the equation, i.e., *σ* = 1/*ρ* = 1/(*dR*) (*σ* is the electrical conductivity, *d* is the thickness of the sample, *R* is the sheet resistance and *ρ* is the electrical resistivity). In addition, a positive correlation between electrical conductivity and EMI shielding effectiveness has been already verified [41]. The maximum *SE*_total_ value reaches up to 29.3 dB (Figure 6b), comparable to or even higher than that of many EMI shielding materials like scoured canvas fabric/polyaniline (13 dB) [42], nickel-plated multiwalled carbon nanotubes/high-density polyethylene composites (12−16 dB) [43], carbonyliron powder-carbon fiber cloth/epoxy resin (12−47 dB) [44], neat carbon nanofiber networks (17−18 dB) [45], d-Ti_3_C_2_T_x_/cellulose nanofiber composite paper (21−26 dB) [46] and carbon nanofiber–graphene nanosheet networks (25−28 dB) [45]. In addition, specific shielding effectiveness (*SSE*) is derived to compare the effectiveness of shielding materials taking into account the density. Mathematically, *SSE* can be obtained by dividing the *SE*_total_ by density of material. In addition, to account for the thickness contribution, absolute effectiveness (*SSE_t_*) is introduced and calculated by dividing the *SSE* by thickness of material. As show in Table 1, the *SSE* and *SSE_t_* values of nano-Cu/CDCFs are about 92 and 4621 dB cm^2^ g^–1^, respectively, comparable with those of these above composites. Furthermore, our synthetic method is relatively simpler and more easily scalable and the composite also has good cost effectiveness and environmental friendliness (free of harmful substances). Inspired by the works reporting Cu-clad carbon fiber nonwoven fabrics [47] and MXene-Graphene-PVDF composite [48] with higher EMI shielding efficiencies, the aim of our future research is to seek better parameters to increase the thickness of Cu composition and also reduce the sputtering time as much as possible, for the sake of preparing more superior EMI shielding property of nano-Cu/CDCFs.

**Figure 7:**
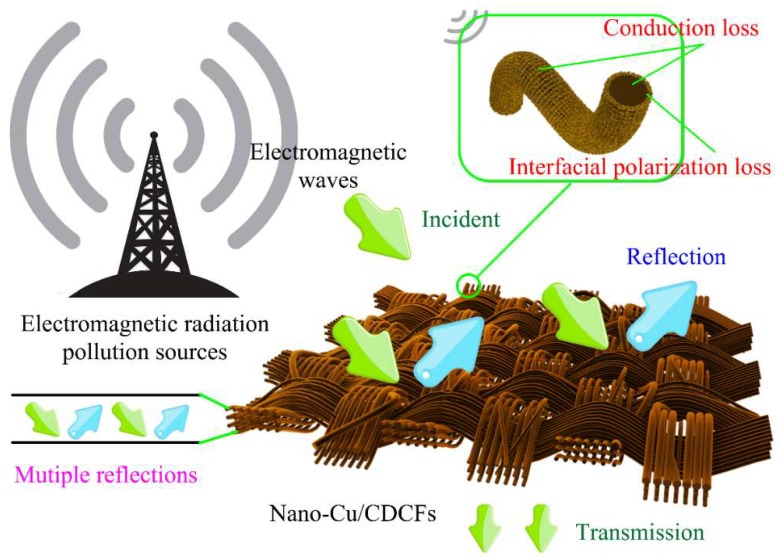
Graphical illustration of the EMI shielding mechanism of the core–shell structured nano-Cu/CDCFs composite.

The synergistic effects of this core–shell structured nano-Cu/CDCFs are responsible for its good EMI shielding property, as illustrated in Figure 7. For CDCFs (core), in the process of electromagnetic wave propagation, the multi-scaled reticulated conductive structure contributes to the occurrence of time-varying electromagnetic-field-induced currents and long-range induced currents. The presence of these currents caused an electric-thermal conversion and rapid decay of massive incident wave [49]. Other effects like dielectric relaxation also caused the attenuation of electromagnetic wave [50]. For nano-Cu (shell), it is well known that copper is one of the most reliable materials in EMI shielding because it is highly effective in attenuating magnetic and electrical waves. In this core–shell material, the existence of interface between the nano-Cu and the CDCFs would cause interfacial polarization loss under an electromagnetic field and the formed conductive Cu layer could induce conduction loss [51,52].

The contribution from *SE*_A_ accounts for 61.1% at 8.2 GHz, much higher than that from *SE*_R_ (38.9%). The higher contribution from *SE*_A_ can be explained by the equations of (4) and (5) [53]:

(4) $$SE_{A}=20d\sqrt{\frac{\mu_{r}\omega \sigma_{AC}}{2}}\cdot \log e,$$

(5) $$SE_{R}=10\log(\frac{\sigma_{AC}}{16\omega \mu_{r}\varepsilon_{0}}),$$

where *d* is the thickness of the shield, *μ_r_* is the magnetic permeability, *ω* is the angular frequency, σ_AC_ is the frequency dependent conductivity and *ε*_0_ is the permittivity of the free space. Obviously, dependence of *SE*_A_ and *SE*_R_ on conductivity and permeability indicates that the material having higher conductivity and magnetic permeability can achieve better absorption properties. This absorption-dominant EMI shielding mechanism of nano-Cu/CDCFs is beneficial to alleviate secondary radiation and considered as a more attractive alternative for the fabrication of electromagnetic radiation protection products.

## 4. Conclusions

An easily-operated and scalable two-step method (pyrolysis and magnetron sputtering) is developed to create a green and core–shell structured composite of nano-Cu/CDCFs. The flexible composite shows numerous alluring properties like excellent hydrophobic property (WCA = 144°), outstanding antibacterial activity against *E. coli* and *S. aureus* (antibacterial ratios of >92%), and good EMI shielding ability with a high *SE*_total_ value of 29.3 dB and absorption-dominant shielding mechanism, due to the physicochemical properties, nano-size effect and synergistic effects of the two components. In conclusion, this multi-purpose eco-friendly biomass-based product is expected to find applications in many fields, e.g., self-cleaning wall cladding, waterproof layer, antibacterial agents and EMI shielding case.

## Figures and Tables

**Table 1:** Comparison of EMI shielding properties of composites.

Composites	Maximum *SE*_total_/dB	*SSE*/dB cm^3^ g^−1^	*SSE_t_*/dB cm^2^ g^−1^	Ref.
Scoured canvas fabric/polyaniline	13	–	–	[42]
Nickel-plated multiwalled carbon nanotubes/high-density polyethylene composites	16	–	–	[43]
Carbonyliron powder-carbon fiber cloth/epoxy resin	47	–	–	[44]
Neat carbon nanofiber networks	18	180	6667	[45]
d-Ti_3_C_2_T_x_/cellulose nanofiber composite paper	25.8	12.4	2647	[46]
Carbon nanofiber–graphene nanosheet networks	28	280	10,370	[45]
Nano-Cu/CDCFs	29.3	92	4621	This work

## References

[1] Han R., Zhang L., Song C., Zhang M., Zhu H., Zhang L. (2010). Characterization of modified wheat straw, kinetic and equilibrium study about copper ion and methylene blue adsorption in batch mode. Carbohydr. Polym..

[2] Li D., Zhu F.Z., Li J.Y., Na P., Wang N. (2013). Preparation and Characterization of Cellulose Fibers from Corn Straw as Natural Oil Sorbents. Ind. Eng. Chem. Res..

[3] Lv S., Fu F., Wang S., Huang J., Hu L. (2015). Novel wood-based all-solid-state flexible supercapacitors fabricated with a natural porous wood slice and polypyrrole. RSC Adv..

[4] Jiao Y., Wan C., Li J. (2017). Scalable synthesis and characterization of free-standing supercapacitor electrode using natural wood as a green substrate to support rod-shaped polyaniline. J. Mater. Sci. Mater. Electron..

[5] Zhou Q., Gong W., Xie C., Yang D., Ling X., Yuan X., Chen S., Liu X. (2011). Removal of Neutral Red from aqueous solution by adsorption on spent cottonseed hull substrate. J. Hazard. Mater..

[6] Chen C., Zhang Y., Li Y., Dai J., Song J., Yao Y., Gong Y., Kierzewski I., Xie J., Hu L. (2017). All-wood, low tortuosity, aqueous, biodegradable supercapacitors with ultra-high capacitance. Energy Environ. Sci..

[7] Wang L., Gao B., Peng C., Peng X., Fu J., Chu P.K., Huo K. (2015). Bamboo leaf derived ultrafine Si nanoparticles and Si/C nanocomposites for high-performance Li-ion battery anodes. Nanoscale.

[8] Khalil H.P.S.A., Bhat A.H., Yusra A.F.I. (2012). Green composites from sustainable cellulose nanofibrils: A review. Carbohydr. Polym..

[9] Yang Q., Fukuzumi H., Saito T., Isogai A., Zhang L. (2011). Transparent cellulose films with high gas barrier properties fabricated from aqueous alkali/urea solutions. Biomacromolecules.

[10] Kim J.-H., Kim J.-H., Choi E.-S., Yu H.K., Kim J.H., Wu Q., Chun S.-J., Lee S.-Y., Lee S.-Y. (2013). Colloidal silica nanoparticle-assisted structural control of cellulose nanofiber paper separators for lithium-ion batteries. J. Power Sources.

[11] Wang Q., Cai J., Zhang L., Xu M., Cheng H., Han C.C., Kuga S., Xiao J., Xiao R. (2013). A bioplastic with high strength constructed from a cellulose hydrogel by changing the aggregated structure. J. Mater. Chem. A.

[12] Wan C., Li J. (2015). Facile synthesis of well-dispersed superparamagnetic γ-Fe_2_O_3_ nanoparticles encapsulated in three-dimensional architectures of cellulose aerogels and their applications for Cr(VI) removal from contaminated water. ACS Sustain. Chem. Eng..

[13] Wan C., Li J. (2016). Cellulose aerogels functionalized with polypyrrole and silver nanoparticles: In-situ synthesis, characterization and antibacterial activity. Carbohydr. Polym..

[14] Lu Y., Liu H., Gao R., Xiao S., Zhang M., Yin Y., Wang S., Li J., Yang D. (2016). Coherent-interface-assembled Ag_2_O-anchored nanofibrillated cellulose porous aerogels for radioactive iodine capture. ACS Appl. Mater. Interfaces.

[15] Moon R.J., Martini A., Nairn J., Simonsen J., Youngblood J. (2011). Cellulose nanomaterials review: Structure, properties and nanocomposites. Chem. Soc. Rev..

[16] Wan C., Li J. (2015). Embedding ZnO nanorods into porous cellulose aerogels via a facile one-step low-temperature hydrothermal method. Mater. Des..

[17] Shi Z., Gao H., Feng J., Ding B., Cao X., Kuga S., Wang Y., Zhang L., Cai J. (2014). In situ synthesis of robust conductive cellulose/polypyrrole composite aerogels and their potential application in nerve regeneration. Angew. Chem. Int. Ed..

[18] Yin Y., Huang R., Zhang W., Zhang M., Wang C. (2016). Superhydrophobic–superhydrophilic switchable wettability via TiO_2_ photoinduction electrochemical deposition on cellulose substrate. Chem. Eng. J..

[19] Zhang X., Lin Z., Chen B., Zhang W., Sharma S., Gu W., Deng Y. (2014). Solid-state flexible polyaniline/silver cellulose nanofibrils aerogel supercapacitors. J. Power Sources.

[20] Kemell M., Pore V., Ritala M., Leskelä M., Lindén M. (2005). Atomic layer deposition in nanometer-level replication of cellulosic substances and preparation of photocatalytic TiO_2_/cellulose composites. J. Am. Chem. Soc..

[21] Korhonen J.T., Hiekkataipale P., Malm J., Karppinen M., Ikkala O., Ras R.H.A. (2011). Inorganic hollow nanotube aerogels by atomic layer deposition onto native nanocellulose templates. ACS Nano.

[22] Kelly P.J., Arnell R.D. (2000). Magnetron sputtering: A review of recent developments and applications. Vacuum.

[23] Wan C., Jiao Y., Liang D., Wu Y., Li J. (2018). A geologic architecture system-inspired micro-/nano-heterostructure design for high-performance energy storage. Adv. Energy Mater..

[24] Sarakinos K., Alami J., Konstantinidis S. (2010). High power pulsed magnetron sputtering: A review on scientific and engineering state of the art. Surf. Coat. Technol..

[25] Alexeeva O.K., Fateev V.N. (2016). Application of the magnetron sputtering for nanostructured electrocatalysts synthesis. Int. J. Hydrog. Energy.

[26] Wang Q., Xiao S., Shi S.Q., Xu S., Cai L. (2019). Self-bonded natural fiber product with high hydrophobic and EMI shielding performance via magnetron sputtering Cu film. Appl. Surf. Sci..

[27] Wan C., Jiao Y., Li J. (2017). A cellulose fibers-supported hierarchical forest-like cuprous oxide/copper array architecture as a flexible and free-standing electrode for symmetric supercapacitors. J. Mater. Chem. A.

[28] Daoud W.A., Xin J.H., Zhang Y.H. (2005). Surface functionalization of cellulose fibers with titanium dioxide nanoparticles and their combined bactericidal activities. Surf. Sci..

[29] Mi L., Licina G.A., Jiang S. (2014). Nonantibiotic-based pseudomonas aeruginosa biofilm inhibition with osmoprotectant analogues. ACS Sustain. Chem. Eng..

[30] Nishiyama Y., Langan P., Chanzy H. (2002). Crystal structure and hydrogen-bonding system in cellulose Iβ from synchrotron x-ray and neutron fiber diffraction. J. Am. Chem. Soc..

[31] Popov V.V., Orlova T.S., Magarino E.E., Bautista M.A., Martínez-Fernández J. (2011). Specific features of electrical properties of porous biocarbons prepared from beech wood and wood artificial fiberboards. Phys. Solid State.

[32] Dandekar A., Baker R.T.K., Vannice M.A. (1998). Characterization of activated carbon, graphitized carbon fibers and synthetic diamond powder using TPD and DRIFTS. Carbon.

[33] Bhushan B., Jung Y.C. (2011). Natural and biomimetic artificial surfaces for superhydrophobicity, self-cleaning, low adhesion, and drag reduction. Prog. Mater. Sci..

[34] Kim M.J., Kim Y.K., Kim K.H., Kwon T.Y. (2011). Shear bond strengths of various luting cements to zirconia ceramic: Surface chemical aspects. J. Dent..

[35] Wan C., Jiao Y., Sun Q., Li J. (2016). Preparation, characterization, and antibacterial properties of silver nanoparticles embedded into cellulose aerogels. Polym. Compos..

[36] Longano D., Ditaranto N., Cioffi N., di Niso F., Sibillano T., Ancona A., Conte A., del Nobile M.A., Sabbatini L., Torsi L. (2012). Analytical characterization of laser-generated copper nanoparticles for antibacterial composite food packaging. Anal. Bioanal. Chem..

[37] Peña M.M.O., Koch K.A., Thiele D.J. (1998). Dynamic regulation of copper uptake and detoxification genes in *Saccharomyces cerevisiae*. Mol. Cell. Biol..

[38] Kang S., Herzberg M., Rodrigues D.F., Elimelech M. (2008). Antibacterial effects of carbon nanotubes: Size does matter!. Langmuir.

[39] Wan C., Jiao Y., Qiang T., Li J. (2017). Cellulose-derived carbon aerogels supported goethite (α-FeOOH) nanoneedles and nanoflowers for electromagnetic interference shielding. Carbohydr. Polym..

[40] Wan C., Li J. (2017). Synthesis and electromagnetic interference shielding of cellulose-derived carbon aerogels functionalized with α-Fe_2_O_3_ and polypyrrole. Carbohydr. Polym..

[41] Li N., Huang Y., Du F., He X., Lin X., Gao H., Ma Y., Li F., Chen Y., Eklund P.C. (2006). Electromagnetic interference (EMI) shielding of single-walled carbon nanotube epoxy composites. Nano Lett..

[42] Akşit A.C., Onar N., Ebeoglugil M.F., Birlik I., Celik E. (2009). Ozdemir, Ismail, Electromagnetic and electrical properties of coated cotton fabric with barium ferrite doped polyaniline film. J. Appl. Polym. Sci..

[43] Yim Y.J., Rhee K.Y., Park S.J. (2016). Electromagnetic interference shielding effectiveness of nickel-plated MWCNTs/high-density polyethylene composites. Compos. Part B Eng..

[44] Hu T., Wang J., Wang J. (2015). Electromagnetic interference shielding properties of carbon fiber cloth based composites with different layer orientation. Mater. Lett..

[45] Song W.-L., Wang J., Fan L.-Z., Li Y., Wang C.-Y., Cao M.-S. (2014). Interfacial engineering of carbon nanofiber–graphene–carbon nanofiber heterojunctions in flexible lightweight electromagnetic shielding networks. ACS Appl. Mater. Interfaces.

[46] Cao W.T., Chen F.F., Zhu Y.J., Zhang Y.G., Jiang Y.Y., Ma M.G., Chen F. (2018). Binary strengthening and toughening of MXene/cellulose nanofiber composite paper with nacre-inspired structure and superior electromagnetic interference shielding properties. ACS Nano.

[47] Lee J., Liu Y., Liu Y., Park S.J., Park M., Kim H.Y. (2017). Ultrahigh electromagnetic interference shielding performance of lightweight, flexible, and highly conductive copper-clad carbon fiber nonwoven fabrics. J. Mater. Chem. C.

[48] Raagulan K., Braveenth R., Jang H., Lee Y.S., Yang C.M., Kim B.M., Moon J.J., Chai K. (2018). Electromagnetic shielding by MXene-Graphene-PVDF composite with hydrophobic, lightweight and flexible graphene coated fabric. Materials.

[49] Song Q., Ye F., Yin X., Li W., Li H., Liu Y., Li K., Xie K., Li X., Fu Q. (2017). Carbon nanotube–multilayered graphene edge plane core–shell hybrid foams for ultrahigh-performance electromagnetic-interference shielding. Adv. Mater..

[50] Micheli D., Vricella A., Pastore R., Marchetti M. (2014). Synthesis and electromagnetic characterization of frequency selective radar absorbing materials using carbon nanopowders. Carbon.

[51] Zhao Z., Zheng W., Yu W., Long B. (2009). Electrical conductivity of poly(vinylidene fluoride)/carbon nanotube composites with a spherical substructure. Carbon.

[52] Zhao B., Park C.B. (2017). Tunable Electromagn. shielding properties of conductive poly(vinylidene fluoride)/Ni chain composite films with negative permittivity. J. Mater. Chem. C.

[53] Ohlan A., Singh K., Chandra A., Dhawan S.K. (2010). Microwave absorption behavior of core−shell structured poly (3,4-ethylenedioxy thiophene)−barium ferrite nanocomposites. ACS Appl. Mater. Interfaces.

